# Bayes Conditional Probability-Based Causation Analysis between Gestational Diabetes Mellitus (GDM) and Pregnancy-Induced Hypertension (PIH): A Statistic Case Study in Harbin, China

**DOI:** 10.1155/2022/2590415

**Published:** 2022-04-22

**Authors:** Dan Diao, Fang Diao, Bin Xiao, Ning Liu, Dan Zheng, Fengjuan Li, Xu Yang

**Affiliations:** ^1^Red Cross Central Hospital (Harbin Obstetrics and Gynecology Hospital Affiliated with Harbin Medical University), Harbin 150001, China; ^2^School of Civil Engineering, Harbin Institute of Technology, Harbin 150090, China

## Abstract

Both gestational diabetes mellitus (GDM) and pregnancy-induced hypertension (PIH) would influence the gestation significantly. However, the causation between these two symptoms remains speculative. 16,404 pregnant women were identified in Harbin, China, in this study. We investigated and evaluated the causal effect of GDM on PIH based on the Bayes conditional probability. The statistical results indicated that PIH might cause GDM, but not vice versa. Also, this case study demonstrated that the decrease temperature might also cause hypertension during pregnancy, and the prevalence rate of GDM increased with age. However, the prevalence of diabetes did not show a remarkable difference in varied areas and ages. This study could provide some essential information that will help to investigate the mechanism for GDM and PIH.

## 1. Introduction

Both gestational diabetes mellitus (GDM) and pregnancy-induced hypertension (PIH) would influence the gestation significantly. However, the causation between these two symptoms remains speculative.

It has been demonstrated that the individuals with diabetes mellitus (including type 1 and 2 diabetes mellitus) would be more likely diagnosed with hypertension than nondiabetics [1] The aortic arteriosclerosis of diabetics would accelerate remarkably [2], and their arterial compliance and elasticity decreased, which would directly cause systolic pressure increase [3]. Meanwhile, the damage of peripheral nerve caused by diabetes might induce microvascular dysfunction, which would also lead to an increase in systolic pressure [4–6].

All these discussions mentioned above were based on the influence of insulin resistance [7, 8]. In the early stage of insulin resistance, hyperinsulinemia would cause reabsorption of sodium by kidney tubules, which cause sympathetic activity frequently [9]. Then, the increased vasoconstriction led to the smooth muscle of small artery proliferation and anastomotic stenosis. The intracellular calcium concentration increased, and the sensitivity of the vasopressor increased. Finally, hypertension would be observed [10].

Otherwise, the mechanism of GDM is different from the other types of diabetes. GDM is a condition defined as any degree of glucose intolerance that starts or is first recognized during pregnancy, and it is characterized by recent hyperglycemia as a consequence of an association between insulin resistance and adequate insulin secretion [11–13]. The influence of GDM on hypertension or PIH has remained unclear.

Bayes conditional probability method provides a means of analyzing the causation between two events only based on prior knowledge of conditions that might be related to the event [14–19]. In this study, we attempt to investigate and evaluate the causal effect of GDM on PIH based on the Bayes decision rule. 16,404 pregnant women were included in this study. By implementing the Bayesian method for epidemiological research [20], the statistical results demonstrated that PIH might cause GDM, but not vice versa. This study could provide some essential information that will help to investigate the mechanism for GDM and PIH.

## 2. Materials and Methods

### 2.1. Bayes Conditional Probability

The events that were diagnosed with PIH and GDM were denoted as *P* and *G*, respectively. The events that were not diagnosed with PIH nor GDM were denoted as *Q* and *H*, respectively. The event that was diagnosed with both PIH and GDM was denoted as *G*∩*P*. Then, the causal effect of GDM on PIH could be analyzed by calculating the probability of event *P* occurring given that *G* is true, i.e., *P*(*P* | *G*). According to the Bayesian conditional probability, it could be given by [21]

(1) $$\begin{array}{c} P\left(P\left|G\right.\right)=\frac{P\left(G\left|P\right.\right)\cdot P\left(P\right)}{P\left(G\right)}, \end{array}$$

where *P*(*G*|*P*) could be obtained based on the conditional probability

(2) $$\begin{array}{c} P\left(G\left|P\right.\right)=\frac{P\left(G\cap P\right)}{P\left(P\right)}. \end{array}$$

*P*(*P*), *P*(*G*), and *P*(*GP*) could be considered as prior probabilities. In this study, since these three prior probabilities all could be obtained by the statistic data, Equation (1) was equivalent to the probability of *P* under condition *G*:

(3) $$\begin{array}{c} P\left(P\left|G\right.\right)=\frac{P\left(G\cap P\right)}{P\left(G\right)}. \end{array}$$

The same procedure could be easily adapted to discuss the causal effect of PIH on GDM. This probability could be given by

(4) $$\begin{array}{c} P\left(G\;|\;P\right)=\frac{P\left(P\;|\;G\right)\cdot P\left(G\right)}{P\left(P\right)}=\frac{P\left(P\cap G\right)}{P\left(P\right)}. \end{array}$$

### 2.2. Case Study on Pregnant Women

To identify cases, 16,404 pregnant women were included in an outpatient setting (hospital outpatient departments of Red Cross Central Hospital) in Harbin, China, between December 22, 2018, and December 28, 2020. All these pregnant women were considered as the total sample in this study. We included all outpatients with a documented diagnosis of pregnancy during about two years to improve diagnostic validity. The date of the first-time pregnancy diagnosis during the study period was assigned as their index date. These pregnant women were aged between 14 and 50.

It should be noticed that the medical testing standards would influence the diagnosis obviously. And the statistic data and the analysis results would then be affected. All the testing standards mentioned in this study followed the manners introduced in [22]. Specifically, the testing and diagnosis method mentioned would be introduced briefly as follow: the GDM would be confirmed according to the oral glucose tolerance test results starting from the 24^th^ to 28^th^ week of gestation. The PIH would be checked according to the blood pressure from the 20^th^ week of gestation. Therefore, the statistical analysis discussion in this study mostly was based on pregnant women in the mid or late trimester of pregnancy.

Besides, the eclampsia would be determined by both hypertension and high urinary protein observed. In the diagnosis issued by the hospital outpatient departments of Red Cross Central Hospital, the eclampsia and PIH would be discussed separately. Thus, the PIH samples discussed in this study did not include those diagnosed with eclampsia.

Also, the ages of these pregnant women and their first diagnosis date were considered which might influence the causation between GDM and PIH. These two factors would be studied and discussed as well.

## 3. Results

By applying Python programming as well as Excel, the data were analyzed, and the results obtained were illustrated as follows. Of these 16,404 pregnant women, 2,540 (15.48%) and 218 (1.33%) were diagnosed with the GDM and PIH, respectively. Meanwhile, 114 (0.69%) had both the GDM and PIH. The probability of *P* under condition *G* is *P*(*P* | *G*) = 4.34%. Relatively, the probability the probability of *G* under condition *P* is *P*(*G* | *P*) = 34.75%.

It should be noted there were very few patients aged less than 20 and more than 43. Therefore, estimates of the prevalence rate were imprecise, and these data were neglected in the following studies and discussions. Under the influence of the age (20-43), *P*(*G* | *P*) and *P*(*P* | *G*) fluctuated in the range of 0 to 14.29% and 0 to 80.00%, respectively. With respect to the influence of the month, *P*(*G* | *P*) and *P*(*P* | *G*) fluctuated in the range of 2.42% to 6.60% and 18.52% to 45.45%, respectively (more details could be found in Table 1 and 2).

**Figure 1:**
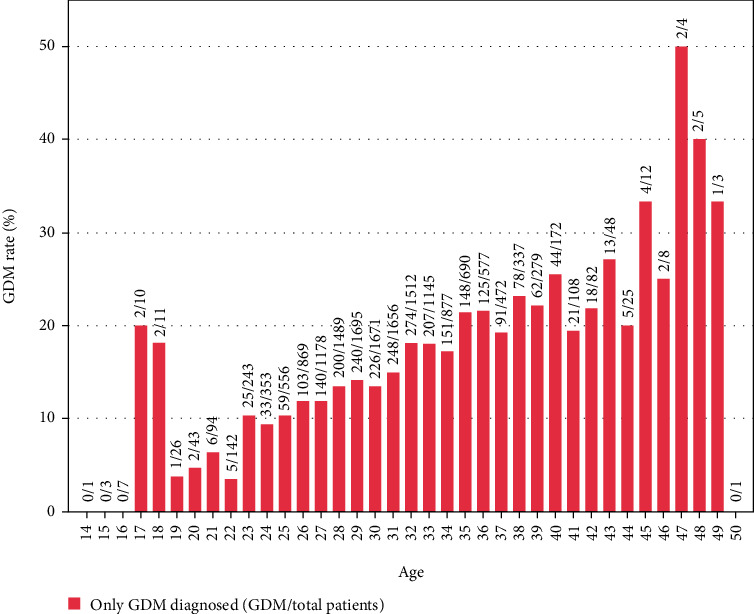
GDM rate distribution histogram with age.

**Figure 2:**
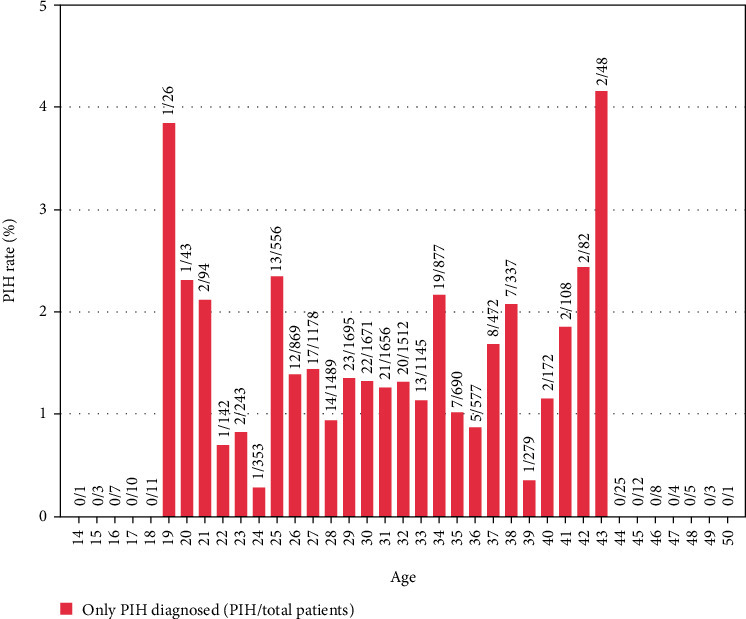
PIH rate distribution histogram with age.

**Figure 3:**
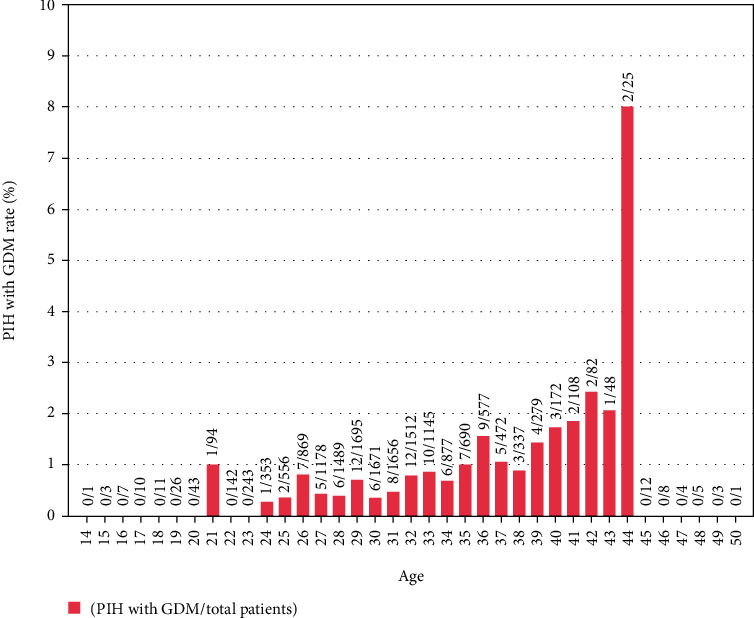
PIH rate distribution histogram with age.

**Figure 4:**
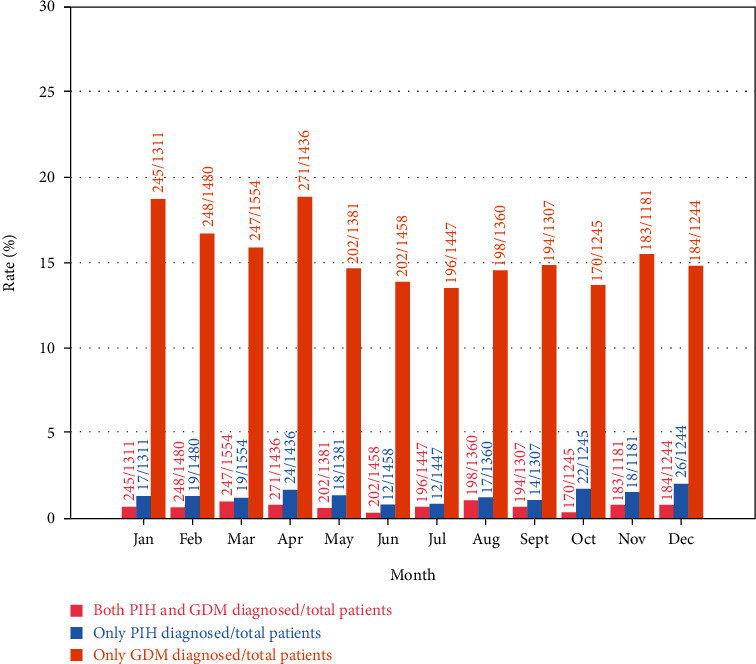
PIH, GDM, and both rate distribution histogram with month.

Figures 1–3 represented the distribution of GDM, PIH, and both by age, respectively.  Figure 4 represented the distribution of GDM, PIH, and both by month.

According to Figures 1–3, it could be observed that the GDM affects 10-25% of pregnancies and PIH affects 1-5%. Moreover, the prevalence rate of GDM increased with age. By contrast, it is not obvious how the patient's age influenced the PIH. In addition, the GDM and PIH were observed relatively less in June and July.

## 4. Discussion

It should be noted that both diabetes combined with pregnancy and GDM would cause blood glucose to increase in pregnancy [23]. The medical record provided by outpatient departments had discriminated against these two conditions. In this study, it could be considered that the patients diagnosed with GDM had normal blood glucose before the pregnancy. By the same logic, the patients diagnosed with PIH could be treated that had normal blood pressure before the pregnancy. Therefore, it could be said that the pregnancy caused the GDM and PIH to some degree. The increased blood glucose or diabetes caused by pregnancy could be considered as exposure and the PIH as an outcome, or vice versa. Based on these assumptions, the following discussions could be drawn:

Firstly, the relationship between GDM and PIH would be discussed. The probability of *P* under condition G (*P*(*P* | *G*) = 4.34%) was obviously smaller than the probability of *G* under condition *P* (*P*(*G* | *P*) = 34.75%). These two probabilities indicated that the PIH might cause the GDM, but the GDM was not likely to cause GDM in the view of statistics.

Secondly, the influence of region on the symptom would be discussed. The studies during 2013-2018 showed that the overall prevalence of GDM was 10-20% according to the IADPSG criteria [24–26]. Several types of research represented that the overall prevalence of PIH was less than 1% [27, 28]. In general, the prevalence of hypertension was higher in cold areas than others [29]. The statistical results induced in this case study identified in the northeast of China demonstrated that the decrease temperature might also cause hypertension during pregnancy. However, the prevalence of diabetes did not show a remarkable difference in varied areas.

Finally, the influence of age and month on GDM and PIH would be discussed. The age might be a notable factor on GDM [30].  Figure 1 and the statistical results represented in Table 1 and 2 demonstrated that older maternal age was significantly associated with risk of GDM. Besides, the lower prevalence of PIH drawn in Figure 3 also demonstrated the influence of temperature on hypertension during pregnancy.

In this study, the sample was analyzed only based on the statistic theory. The pathological mechanism would be discussed in our future works.

## 5. Conclusions

This study analyzed the causal relationship between the GDM and PIH based on the Bayes conditional probability. The following conclusions could be drawn:

1. The smaller probability of *P* under condition G (*P*(*P* | *G*) = 4.34%) and the larger probability of *G* under condition P (*P*(*G* | *P*) = 34.75%) indicated that the PIH might cause the GDM. However, the GDM was not likely to cause PIH in the view of statistics
2. The statistical results induced in this case study identified in the northeast of China demonstrated that the decrease temperature might also cause hypertension during pregnancy. However, the prevalence of diabetes did not show a remarkable difference in varied areas
3. The statistic results indicated that older maternal age was significantly associated with the risk of GDM

This study contributes to the characterization of the prevalence rate of GDM and PIH, as well as the mechanism of these conditions during pregnancy.

## Figures and Tables

**Table 1:** Statistical results distributed by month.

Month	Amount	Only GDM	*P*(*G*)	Only PIH	*P*(*P*)	Both	*P*(*PG*)	*P*(*P* | *G*)	*P*(*G* | *P*)
Jan	1311	245	18.69%	17	1.30%	9	0.69%	3.54%	34.62%
Feb	1480	248	16.76%	19	1.28%	9	0.61%	3.50%	32.14%
Mar	1554	247	15.89%	19	1.22%	15	0.97%	5.73%	44.12%
Apr	1436	271	18.87%	24	1.67%	11	0.77%	3.90%	31.43%
May	1381	202	14.63%	18	1.30%	8	0.58%	3.81%	30.77%
Jun	1458	202	13.85%	12	0.82%	5	0.34%	2.42%	29.41%
Jul	1447	196	13.55%	12	0.83%	10	0.69%	4.85%	45.45%
Aug	1360	198	14.56%	17	1.25%	14	1.03%	6.60%	45.16%
Sept	1307	194	14.84%	14	1.07%	9	0.69%	4.43%	39.13%
Oct	1245	170	13.65%	22	1.77%	5	0.40%	2.86%	18.52%
Nov	1181	183	15.50%	18	1.52%	9	0.76%	4.69%	33.33%
Dec	1244	184	14.79%	26	2.09%	10	0.80%	5.15%	27.78%
Total	16404	2540	15.48%	218	1.33%	114	0.69%	4.30%	34.34%

**Table 2:** Statistical results distributed by age.

Month	Amount	Only GDM	*P*(*G*)	Only PIH	*P*(*P*)	Both	*P*(*PG*)	*P*(*P* | *G*)	*P*(*G* | *P*)
14	1	0	0.00%	0	0.00%	0	0.00%	—	—
15	3	0	0.00%	0	0.00%	0	0.00%	—	—
16	7	0	0.00%	0	0.00%	0	0.00%	—	—
17	10	2	20.00%	0	0.00%	0	0.00%	0.00%	—
18	11	2	18.18%	0	0.00%	0	0.00%	0.00%	—
19	26	1	3.85%	1	3.85%	0	0.00%	0.00%	0.00%
20	43	2	4.65%	1	2.33%	0	0.00%	0.00%	0.00%
21	94	6	6.38%	2	2.13%	1	1.06%	14.29%	33.33%
22	142	5	3.52%	1	0.70%	0	0.00%	0.00%	0.00%
23	243	25	10.29%	2	0.82%	0	0.00%	0.00%	0.00%
24	353	33	9.35%	1	0.28%	1	0.28%	2.94%	50.00%
25	556	59	10.61%	13	2.34%	2	0.36%	3.28%	13.33%
26	869	103	11.85%	12	1.38%	7	0.81%	6.36%	36.84%
27	1178	140	11.88%	17	1.44%	5	0.42%	3.45%	22.73%
28	1489	200	13.43%	14	0.94%	6	0.40%	2.91%	30.00%
29	1695	240	14.16%	23	1.36%	12	0.71%	4.76%	34.29%
30	1671	226	13.52%	22	1.32%	6	0.36%	2.59%	21.43%
31	1656	248	14.98%	21	1.27%	8	0.48%	3.13%	27.59%
32	1512	274	18.12%	20	1.32%	12	0.79%	4.20%	37.50%
33	1145	207	18.08%	13	1.14%	10	0.87%	4.61%	43.48%
34	877	151	17.22%	19	2.17%	6	0.68%	3.82%	24.00%
35	690	148	21.45%	7	1.01%	7	1.01%	4.52%	50.00%
36	577	125	21.66%	5	0.87%	9	1.56%	6.72%	64.29%
37	472	91	19.28%	8	1.69%	5	1.06%	5.21%	38.46%
38	337	78	23.15%	7	2.08%	3	0.89%	3.70%	30.00%
39	279	62	22.22%	1	0.36%	4	1.43%	6.06%	80.00%
40	172	44	25.58%	2	1.16%	3	1.74%	6.38%	60.00%
41	108	21	19.44%	2	1.85%	2	1.85%	8.70%	50.00%
42	82	18	21.95%	2	2.44%	2	2.44%	10.00%	50.00%
43	48	13	27.08%	2	4.17%	1	2.08%	7.14%	33.33%
44	25	5	20.00%	0	0.00%	2	8.00%	28.57%	100.00%
45	12	4	33.33%	0	0.00%	0	0.00%	0.00%	—
46	8	2	25.00%	0	0.00%	0	0.00%	0.00%	—
47	4	2	50.00%	0	0.00%	0	0.00%	0.00%	—
48	5	2	40.00%	0	0.00%	0	0.00%	0.00%	—
49	3	1	33.33%	0	0.00%	0	0.00%	0.00%	—
50	1	0	0.00%	0	0.00%	0	0.00%	—	—
Total	16404	2540	15.48%	218	1.33%	114	0.69%	4.30%	34.34%

## Data Availability

No data were used to support this study.
